# Role of calcineurin (CN) in kidney glomerular podocyte: CN inhibitor ameliorated proteinuria by inhibiting the redistribution of CN at the slit diaphragm

**DOI:** 10.14814/phy2.12679

**Published:** 2016-03-23

**Authors:** Ayako Wakamatsu, Yoshiyasu Fukusumi, Eriko Hasegawa, Masayuki Tomita, Toru Watanabe, Ichiei Narita, Hiroshi Kawachi

**Affiliations:** ^1^ Department of Cell BiologyInstitute of NephrologyNiigata University Graduate School of Medical and Dental SciencesNiigataJapan; ^2^ Division of Clinical Nephrology and RheumatologyNiigata University Graduate School of Medical and Dental SciencesNiigataJapan; ^3^ Department of PediatricsNiigata City General HospitalNiigataJapan

**Keywords:** calcineurin, proteinuria, podocyte, slit diaphragm

## Abstract

Although calcineurin (CN) is distributed in many cell types and functions in regulating cell functions, the precise roles of CN remained in each type of the cells are not well understood yet. A CN inhibitor (CNI) has been used for steroid‐resistant nephrotic syndrome. A CNI is assumed to ameliorate proteinuria by preventing the overproduction of T‐cell cytokines. However, recent reports suggest that CNI has a direct effect on podocyte. It is accepted that a slit diaphragm (SD), a unique cell–cell junction of podocytes, is a critical barrier preventing a leak of plasma protein into urine. Therefore, we hypothesized that CNI has an effect on the SD. In this study, we analyzed the expression of CN in physiological and in the nephrotic model caused by the antibody against nephrin, a critical component of the SD. We observed that CN is expressed at the SD in normal rat and human kidney sections and has an interaction with nephrin. The staining of CN at the SD was reduced in the nephrotic model, while CN activity in glomeruli was increased. We also observed that the treatment with tacrolimus, a CNI, in this nephrotic model suppressed the redistribution of CN, nephrin, and other SD components and ameliorated proteinuria. These observations suggested that the redistribution and the activation of CN may participate in the development of the SD injury.

## Introduction

Calcineurin (CN), a Ca^2+^ and calmodulin‐dependent protein serine/threonine phosphatase, is a heterodimeric protein consisting of a catalytic subunit calcineurin A (CN‐A) and a Ca^2+^‐binding subunit calcineurin B (CN‐B). CN is known to be widely distributed in many cell types. The highest level of distribution of CN is found in the brain, and a high level of distribution of CN was observed in the heart, lungs, liver, and lymphocytes. CN is reported to play an important role in the functioning of these cells (Rusnak and Mertz 2000; Shibasaki et al. 2002). It is reported that CN is also highly distributed in the kidneys (Rusnak and Mertz 2000; Shibasaki et al. 2002). However, the precise role of CN in the kidneys is not yet fully understood.

CN inhibitors (CNIs) such as tacrolimus and cyclosporine A are the most widely used immunosuppressive agents. It is understood that CNIs decreased the production of cytokines such as IL‐2 and IL‐4 (O'Keefe et al. 1992; Bram et al. 1993; Azzi et al. 2013), by inhibiting the activation of a nuclear factor of an activated T cell (NFAT), a substrate of CN in T cells. In the nephrology field, because it has been understood that several types of nephrotic syndrome are caused by dysregulation of the T‐cell function (Shalhoub 1974), CNIs have been used for patients with steroid‐resistant nephrotic syndrome. It has been assumed that CNI ameliorated proteinuria by preventing the overproduction of T‐cell cytokines. However, it is known that the effect of CNI on proteinuria does not coincide with its immunosuppressive effect and that CNI reduces proteinuria in patients and the experimental model of Alport syndrome, a nonimmunological disease (Chen et al. 2003; Charbit et al. 2007). Sharma et al. demonstrated that CNI has a direct protective effect on glomeruli with their in vitro assay measuring glomerular albumin permeability (Sharma et al. 1996). It is also reported that the activation of NFAT in kidney glomerular epithelial cells (podocytes) may be a critical pathogenic event in focal segmental glomerulosclerosis (Wang et al. 2010; Nijenhuis et al. 2011) and that CNI ameliorated the dysfunction of podocytes by inhibiting the activation of NFAT (Spurney 2014). It has also been reported that the activation of CN leads to podocyte injury without the involvement of NFAT (Faul et al. 2008; Li et al. 2014; Spurney 2014). Faul et al. showed that synaptopodin, an actin‐associated protein of podocytes, was dephosphorylated by CN in the nephrotic state and that the dephosphorylated synaptopodin, which is easy to be degradeted, participated in the development of podocyte injury and the consequent proteinuria (Faul et al. 2008). Then, they showed that CNI ameliorated podocyte injury by preventing the dephosphorylation of synaptopodin. Another recent report showed that CNI stabilized podocyte cytoskeleton by upregulating expression of cofilin, which was independent of its effect on synaptopodin (Li et al. 2014). All of these reports indicated that CNI has a direct effect on podocytes. However, the precise pharmacological mechanism how CNI ameliorates proteinuria is unclear.

Although the precise pathogenic mechanisms of proteinuria is not fully understood yet, several studies in a past decade indicated that a slit diaphragm (SD), a unique cell–cell junction of podocytes, is a critical barrier preventing a leak of plasma protein into urine (Pavenstadt et al. 2003), and that the dysregulation of the barrier function of the SD is involved in the development of several types of human nephritic syndromes. Therefore, we hypothesized that CN plays a role in regulating the SD function and that CNI has an effect on the SD.

In this study, we analyzed the expression and the localization of CN in podocytes in physiological and the proteinuric states with the antibody recognizing an endogenous form of CN (Talmadge et al. 2004). The expression of CN was detected at the SD in normal rat and human kidney sections. The staining of CN at the SD was clearly decreased in the nephrotic model caused by the injection with the antibody against nephrin, a critical molecule of the SD. On the other hand, the phosphatase activity of CN in glomeruli was increased in the nephrotic model. In this study, we also showed that tacrolimus, a CNI, ameliorated proteinuria in this nephrotic model. Based on these observations, we proposed that the endogenous inactive form of CN has a role in maintaining the barrier function of the SD and that a shift to an active form of CN‐A is involved in the development of the SD injury.

## Materials and Methods

### Animals

Specific pathogen‐free, 6‐week‐old female Wistar rats (Charles River, Japan; Atsugi, Japan) weighing 150–170 g were used. All animal experiments conformed to the National Institutes of Health Guide for the Care and Use of Laboratory Animals. All procedures for the present study were approved by the Animal Committee at Niigata University School of Medicine, and all animals were treated according to the Niigata University guidelines for animal experimentation.

### Cultured podocytes

A conditionally immortalized mouse podocyte cell line was kindly donated by Dr. Peter Mundel (Albert Einstein College of Medicine, Bronx, NY). Cultivation of the cell was conducted as described previously (Mundel et al. 1997).

### Induction of anti‐nephrin antibody‐induced nephropathy

Anti‐nephrin antibody‐induced nephropathy was prepared by an intravenous injection with 10 mg/rat anti‐nephrin monoclonal antibody (mAb 5‐1‐6) (Orikasa et al. 1988; Topham et al. 1999; Kawachi et al. 2000). A total of 18 rats were injected, and six rats each were killed 1 h, 24 h, and 5 days after injection. Twenty‐four‐hour urine samples were collected 5 days after injection. Urinary protein concentrations were measured by colorimetric assay with Protein Assay Reagent (Bio‐Rad, Hercules, CA) using bovine serum albumin as a standard. The average value of proteinuria (±SD) of six rats on day 5 after the antibody injection was 72.0 ± 7.9 mg/day. The rats were killed, and kidneys were removed. The materials were used for immunofluorescence, RT‐PCR, and Western blot. Small tissue samples were used for immunofluorescence studies. To prepare two sets of glomerular RNA for each time point, glomeruli were isolated from the remaining kidney tissue pooled from three rats each by a sieve method, and glomerular RNA was prepared with Trizol (Life Technologies, Inc., Gaithersburg, MD).

### Effect of tacrolimus in anti‐nephrin antibody‐induced nephropathy

Twelve rats were injected with the anti‐nephrin monoclonal antibody and treated with 0.3 mg/kg BW of tacrolimus (FK506) or placebo daily from 5 days before induction of anti‐nephrin antibody‐induced nephropathy (*n* = 6) and then were killed on day 5 after the antibody injection. The kidneys from each animal were cut into portions and used for preparation of glomerular RNA or for the IF study. Six rats injected with RVG1, an irrelevant murine IgG1, were treated with 0.3 mg/kg BW of tacrolimus daily from 5 days before RVG1 injection and were killed on day 5. Twenty‐four‐hour urine samples were collected on days 0, 1, 3, and 5 after injection. Six rats were injected with anti‐nephrin antibody and treated with tacrolimus or saline as described above and killed at 1 h after the antibody injection. Tacrolimus (FK506) and placebo were kindly provided by Astellas Pharmaceutical Co., Ltd. (Osaka, Japan).

### Immunohistochemical study with anti‐CN‐A antibody

Immunohistochemical studies were performed basically according to the method previously reported (Kawachi et al. 2000; Ito et al. 2002; Han et al. 2003). A rabbit anti‐calcineurin pan A antibody was purchased from Millpore, MA, USA. The 3‐*μ*m‐thick frozen sections were fixed with acetone for 1 min at room temperature, incubated at 4°C overnight with the anti‐CN‐A antibody, and stained with FITC‐conjugated swine anti‐rabbit Igs (DAKO, Glostrup, Denmark) at 37°C for 30 min. The anti‐CN‐A antibody recognized both CN‐A‐*α* and CN‐A‐*β*. The sections were observed with immunofluorescence microscopy (BX‐50; Olympus, Tokyo). The specificity of the anti‐CN‐A antibody was confirmed by preabsorption analysis with the GST‐fusion protein of CN‐A. To evaluate the CN‐A staining in the rat glomeruli, the staining was graded semiquantitatively as follows: continuous staining of >75% was score 4; 75–50% was score 3; 50–25% was score 2; and 25–0% was score 1. A score was assigned to each glomerulus, and 30 glomeruli of each rat were analyzed. The data are shown as mean ± SD of six rats (Nakatsue et al. 2005).

### Characterization of the anti‐CN‐A antibody

Characteristics of the anti‐CN‐A antibody used in this study were analyzed by Western blot analyses with the GST‐fusion proteins of full length and the autoinhibitory domain of CN. GST‐fusion proteins were prepared with a vector (pGEX‐6P; Amersham Pharmacia Biotech, Uppsala, Sweden). The full‐length fusion protein was prepared according to the manufacture's manual.

### Dual‐labeling IF study

The dual‐labeling immunofluorescence study was performed basically according to the method of the previous report (Han et al. 2003; Hashimoto et al. 2007; Saito et al. 2011). The following antibodies were used as the glomerular cell markers, mouse anti‐rat endothelial cell antigen‐1 (RECA‐1) antibody (Serotec, Oxford, UK), mouse monoclonal antibody to Thy1.1, a mesangial cell marker (mAb 1‐22‐3), mouse monoclonal antibody to synaptopodin, a podocyte marker (Progen, Heidelberg, Germany), mouse monoclonal antibody to nephrin, an SD marker (mAb 5‐1‐6), mouse monoclonal antibody to ZO‐1, an SD marker (Zymed Laboratories, San Francisco, CA), mouse monoclonal antibody to podocalyxin, a podocyte apical marker (Han et al. 2003), mouse monoclonal antibody to *α*3‐integrin, and a podocyte basal marker (Santa Cruz, CA). FITC‐conjugated swine anti‐rabbit Igs (DAKO) and tetramethylrhodamine isothiocyanate (TRITC)‐conjugated goat anti‐mouse IgG1 (Southern Biotechnology Associated) were used as secondary antibodies.

### Reverse transcription‐polymerase chain reaction (RT‐PCR)

A semiquantitative RT‐PCR analysis with glomerular RNA was performed basically according to the method described previously (Kawachi et al. 2000; Hashimoto et al. 2007). The specific primers were designed for isoforms of CN‐A‐*α* and CN‐A‐*β*: as primers for rat CN‐A‐*α* (sense, 5′‐AGTAACTTTCGAGCCAGCCC‐3′; antisense, 5′‐CAACGCGACACTTTCTTCCAG‐3′) and for mouse (sense, 5′‐CAGTTGAGGCTATTGAGGCTG‐3′; antisense, 5′‐CACGGATCTCAGAAAGCACA‐3′) and as primers for rat CN‐A‐*β* (sense, 5′‐AGCAAGCTGGTTTCAATTCCC‐3′; antisense 5′‐CTTCCTCCACTGGAATTTGC‐3′) and for mouse (sense 5′‐CCCTCTGACGCCAACCTTAAAC‐3′; antisense 5′‐TAGTGCTGCGACTGTAAACG‐3′). For quantification, the band intensity was determined by image analysis using BIO‐RAD Gel Doc^TM^ EZ Imager System and densitometry software, Image Lab 3.0 (Bio‐Rad). The results were corrected for the amount of mRNA in the sample by dividing by the intensity of the internal control glyceraldehydes‐3‐phosphate dehydrogenase (GAPDH). Real‐time RT‐PCR was performed basically according to the method previously reported (Miyauchi et al. 2006).

### Western blot analysis

Western blot analysis was performed basically according to the method described previously (Kawachi et al. 2003; Suzuki et al. 2015). In brief, rat glomeruli was solubilized with sodium dodecyl sulfate (SDS)‐polyacrylamide gel electrophoresis (PAGE) sample buffer (2% SDS, 10% glycerol, 5% mercaptoethanol in 62.5 mmol/L Tris‐HCI [pH6.8]) with protease inhibitors. The solubilized material was subjected to a polyvinylidene fluoride transfer membrane (Pall Corporation, Pensacola, FL). After exposure to the primary antibodies, alkaline phosphatase‐conjugated secondly antibodies were used. The reaction was developed with an alkaline phosphatase chromogen kit (Biomedica, Foster City, CA).

### Immunoprecipitation assays

Immunoprecipitation was performed basically according to the method previously reported (Hashimoto et al. 2007; Otaki et al. 2008). In brief, glomerular lysate solubilized with 1% Triton X‐100 with a protease inhibitor cocktail (Bio‐Rad) was incubated with an anti‐CN‐A antibody or normal rabbit serum at 4°C overnight and precipitated with Dynabeads Protein G (Invitrogen, Carlsbad, CA). The tube was placed in the magnet, and the supernatant was removed. The Dynabeads–antibody–antigen complex was washed five times with PBS containing 0.1% Triton X‐100, and then the antigen was eluted with the SDS‐PAGE sample buffer. The elution fractions were separated by SDS‐PAGE followed by immunoblotting with a rabbit anti‐nephrin antibody or a rabbit anti‐ZO‐1 antibody. The antigen of another tubes was eluated with the SDS‐PAGE sample buffer without mercaptoethanol. The elution fractions were separated by SDS‐PAGE followed by immunoblotting with a rabbit anti‐podocin antibody. Alkaline phosphatase‐conjugated antibody was used as a second antibody.

### Duolink in situ assay

A Duolink in situ assay, which can detect a protein–protein interaction (Soderberg et al. 2006), was performed with Duolink in situ PLA probes purchased from Olink Bioscience, Uppsala, Sweden. The principals of this assay are as follows: two primary antibodies raised in different species were used. Species‐specific secondary antibodies, called PLA probes, with a unique, short DNA strand bind to the primary antibodies. When the PLA probes are in close proximity (<40 nm), the DNA strands can interact. After amplification, the DNA circle is detected with a fluorescence microscope. The analysis was performed according to the method previously reported (Fukusumi et al. 2015). Briefly, rat kidney sections were incubated with rabbit anti‐CN‐A and mouse anti‐nephrin, and the interaction was analyzed with Duolink in situ PLA probe anti‐rabbit PLUS and Duolink in situ PLA probe anti‐mouse MINUS. The interaction was analyzed with Duolink in situ PLA probe anti‐rabbit PLUS and Duolink in situ PLA probe anti‐goat MINUS.

### CN activity assay

The phosphatase activity of CN in glomerular lysates was evaluated with the Calcineurin Cellular Activity Assay kit (Enzo Life Sciences, Farmingdale, NY) according to manufacturer's instructions.

### Human kidney specimens

The kidney specimens were obtained from a patient with MCNS treated at the Department of Pediatrics, Niigata City General Hospital. After the routine studies had been performed, the samples were stored at −30°C until further use. As a control, the normal part of the kidney obtained with nephrectomy was used. This study was approved by the ethics committee of the Niigata City General Hospital. Informed consent was obtained from the patient.

### Statistical analysis

Statistical significance was evaluated with the Mann–Whitney *U*‐test and Student's *t*‐test. All values are expressed as mean ± SD. Data were analyzed with GraphPad Prism 5.0 software (GraphPad Software, San Diego, CA) for Windows.

## Results

### Expression of CN‐A in glomeruli

**Figure 1:**
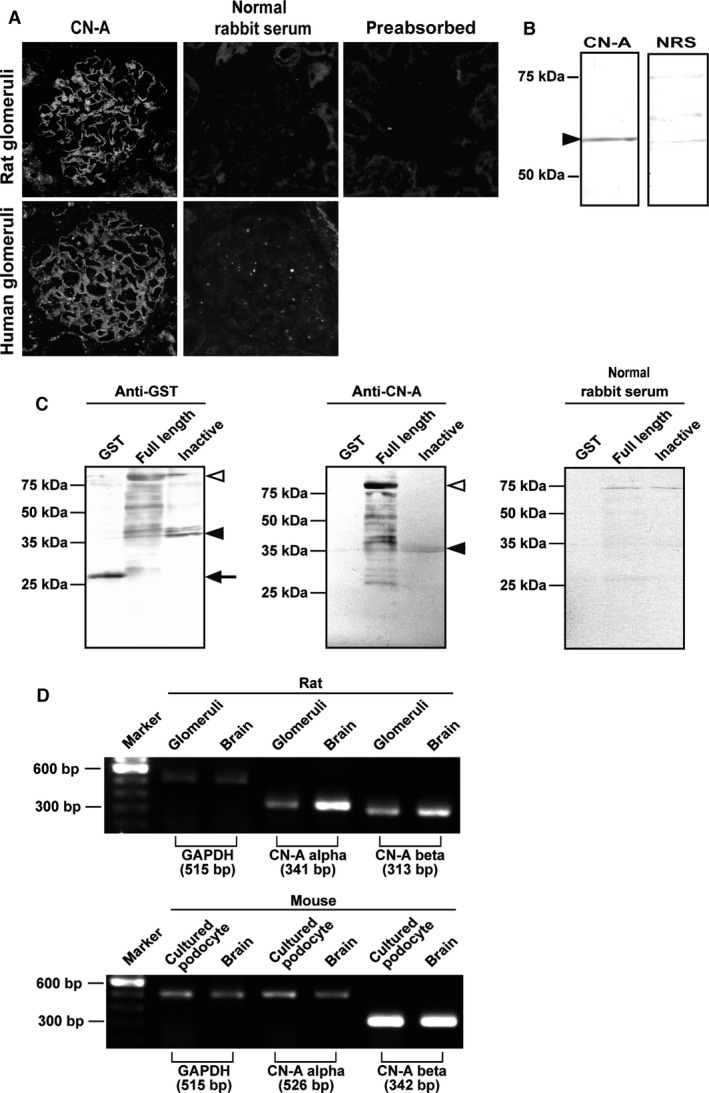
Expression of CN‐A in podocytes in normal glomeruli. (A) Clear positive staining along the capillary wall in the glomeruli of a normal rat kidney section was detected with the rabbit anti‐CN‐A antibody. No specific staining was observed in normal rabbit serum. No positive staining was detected in the antibody preabsorbed with the GST‐fusion protein of full‐length CN‐A, showing that the antibody is specific to CN‐A. Similar staining of CN‐A was detected in the human glomeruli. No staining was observed in the normal rabbit serum in kidney section. (B) A positive band of approximately 60 kDa was detected in a normal rat glomerular lysate with an anti‐CN‐A antibody. No bands were detected with the normal rabbit serum. (C) The antibody recognizes the GST‐fusion protein of full‐length CN‐A. A weak band was detected with the antibody in the GST‐fusion protein of the autoinhibitory domain of CN‐A. (D) mRNA expressions of both CN‐A‐*α* and CN‐A‐*β* were detected in normal rat glomeruli (upper panel) and in the murine‐cultured podocyte (lower panel) (brain, cerebrum sample).

The staining with the rabbit anti‐CN‐A antibody was restricted in the glomeruli in kidney section, and it was found to be a linear pattern along the capillary loop. The representative finding is shown in Figure 1A. No specific staining was observed in normal rabbit serum. No positive staining was detected with the antibody preabsorbed with the GST‐fusion protein of full‐length CN‐A, indicating that the staining is specific to CN‐A. Similar staining along the glomerular capillary wall was also observed in the human kidney section. A positive band around 60 kDa, which corresponds to the full length of CN‐A, was detected with the anti‐CN‐A antibody in the normal rat glomerular lysate (Fig. 1B). The specificity of the anti‐CN‐A antibody was also confirmed by the Western blot analyses with GST‐fusion proteins of CN‐A. The antibody recognizes the fusion proteins of full‐length CN‐A. A weak band was detected with the antibody in the fusion protein of the autoinhibitory domain of CN‐A (Fig. 1C). mRNA expressions of both CN‐A‐*α* and CN‐A‐*β* were detected in glomeruli and mouse‐cultured podocytes (Fig. 1D).

### CN‐A was colocalized with SD molecules

**Figure 2:**
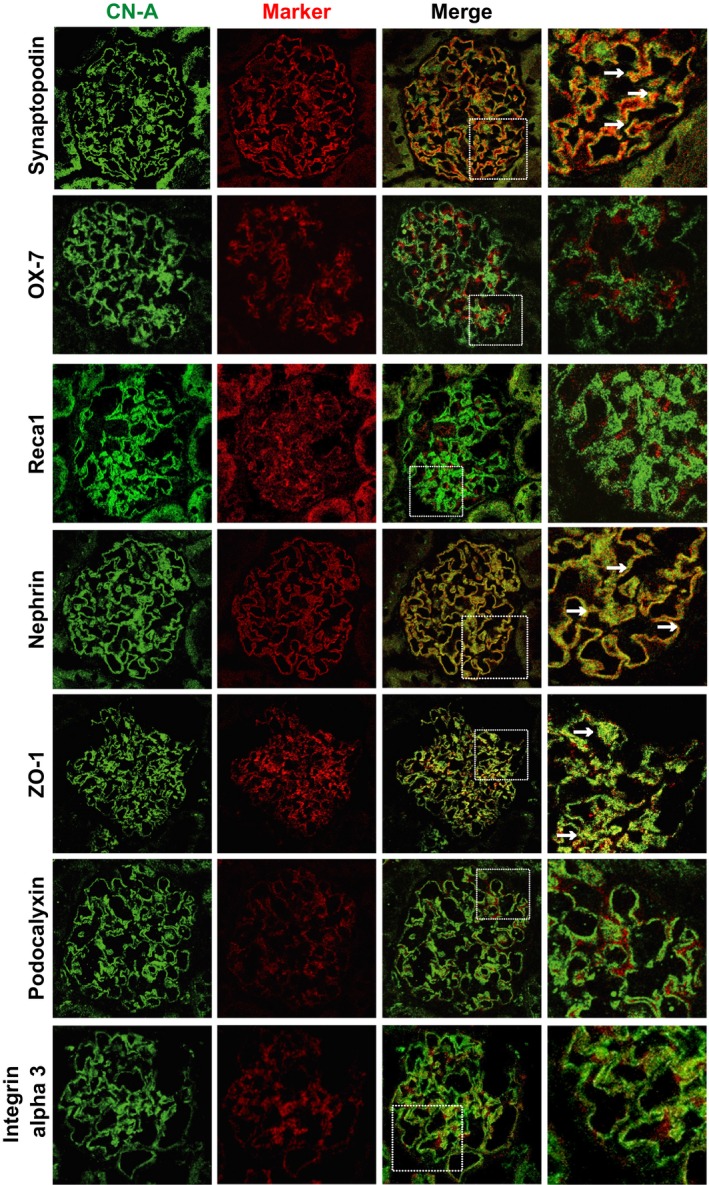
Dual‐labeling IF findings of CN‐A (green) with glomerular cell markers (red). Representative findings of the CN‐A (green), glomerular cell markers (red), and merge are shown. The staining of CN‐A has close proximity with the synaptopodin staining. Some portions of the CN‐A staining were costained with synaptopodin (arrows). The staining of CN‐A was clearly apart from the stainings of OX‐7 and RECA‐1. Major portions of the CN‐A stainings were costained with nephrin and ZO‐1, markers of the SD (arrows). The staining was apart from that of podocalyxin, an apical surface marker, and *α*3‐integrin, a basal surface marker. These observations indicated that the CN‐A was mainly localized at the SD area.

To analyze the precise localization of the CN‐A detected with the antibody, a dual‐labeling immunofluorescence study was carried out with the following glomerular cell markers: (1) the endothelial cell marker RECA‐1; (2) the mesangial cell marker OX‐7; and (3) the podocyte‐marker synaptopodin. The representative findings are shown in Figure 2. The staining of CN‐A has close proximity with the podocalyxin staining. The CN‐A staining was apart from the stainings of RECA‐1 and OX‐7, and no stainings colocalized with these markers were detected, indicating that the full‐length CN‐A was restrictedly expressed in the podocyte. Then, to analyze the subcellular localization of the CN‐A in podocytes, the dual‐labeling IF study with podocyte markers was performed. Major portions of the CN‐A were costained with nephrin and ZO‐1, markers for the SD. The staining was apart from that of podocalyxin, an apical surface marker, and *α*3‐integrin, a basal surface marker. These observations indicated that the CN‐A was colocalized with SD molecules.

### CN‐A has an interaction with nephrin

**Figure 3:**
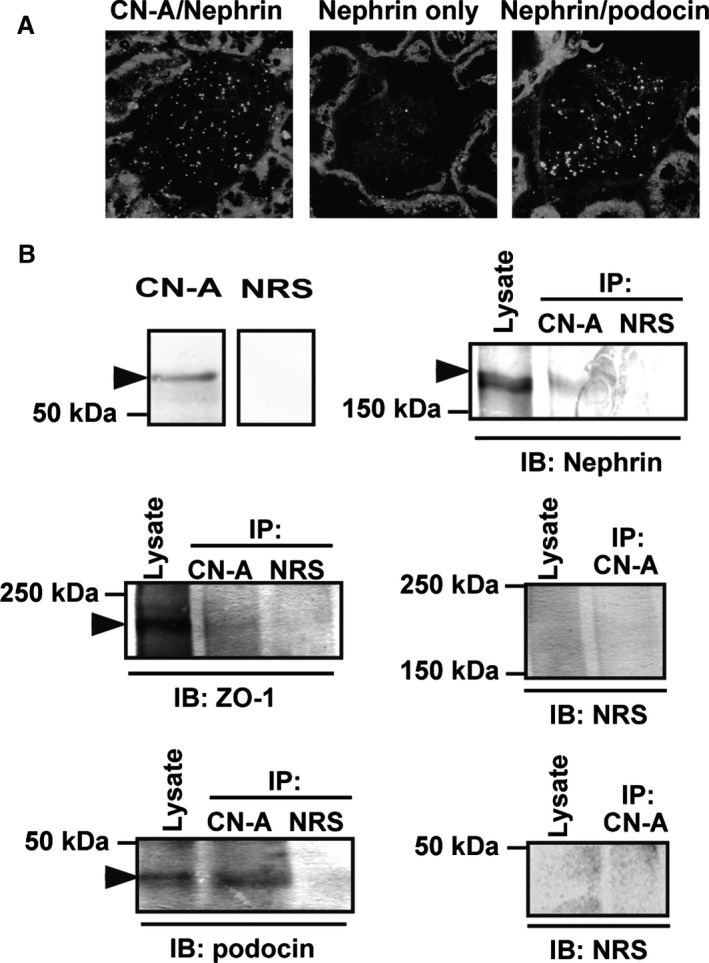
CN‐A has an interaction with nephrin. (A) The positive spots were detected with the Duolink technique in a normal rat kidney section incubated with the antibodies for the CN‐A and nephrin, indicating that these molecules located with close proximity, within 40 nm. (B) Double bands of nephrin were detected in whole lysates of the glomeruli, and an upper band was densely detected in the anti‐CN‐A precipitate (arrowhead). ZO‐1 and podocin were also detected in the anti‐CN‐A precipitate. These observations indicated that the CN‐A has an interaction with the SD components (IP, immunoprecipitate; IB, immunoblot; NRS, normal rabbit serum).

Because the colocalization of CN‐A and nephrin was observed, we examined whether CN‐A has an interaction with nephrin using a Duolink in situ assay and immunoprecipitate assay with normal adult rat materials. In the Duolink assay with the antibodies for CN‐A and nephrin, the red spots were detected in normal rat kidney sections, indicating that these molecules located with close proximity, within 40 nm (Fig. 3A).

A specific nephrin band of 180 kDa was detected in the precipitate with the anti‐CN‐A antibody of the normal rat glomerular lysate (Fig. 3B). Double bands of nephrin were detected in the whole lysate of the glomeruli, and an upper band, which corresponds to nephrin localized at the SD, was densely detected in the anti‐CN‐A precipitate. Not only nephrin but also other SD components, ZO‐1 and podocin, were detected in the anti‐CN‐A precipitate. These observations indicate that CN‐A has an interaction with the SD components.

### Expression of CN‐A in developing glomeruli

**Figure 4:**
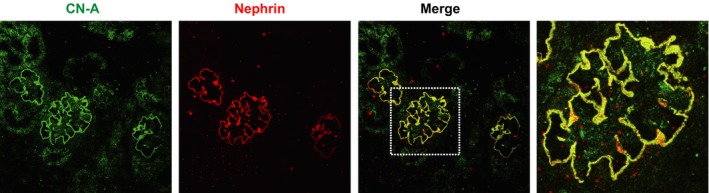
Expression of CN‐A in developing glomeruli. Clear CN‐A staining was detected in a maturing podocyte, and the staining was restrictedly detected at the basal side of the podocyte as a continuous fine granular pattern. The staining of the CN‐A in the maturing podocyte completely coincided with that of nephrin.

To investigate the developmental expression of CN‐A, the dual‐labeling study with the anti‐nephrin antibody was performed in neonatal rat kidney sections. The CN‐A staining in the presumptive podocyte was first detected at the late S‐shaped body stage, when nephrin first appeared. The clear staining was detected at the early capillary loop stage. The staining of CN‐A became clearer with the glomerular stage advancing. The staining was restricted at the basal side of the podocyte as a continuous fine granular pattern in the maturing podocyte. The CN‐A staining in the developing glomeruli completely coincided with that of nephrin. The findings are shown in Figure 4.

### Expression and activity of CN‐A are altered in anti‐nephrin antibody‐induced nephropathy

**Figure 5:**
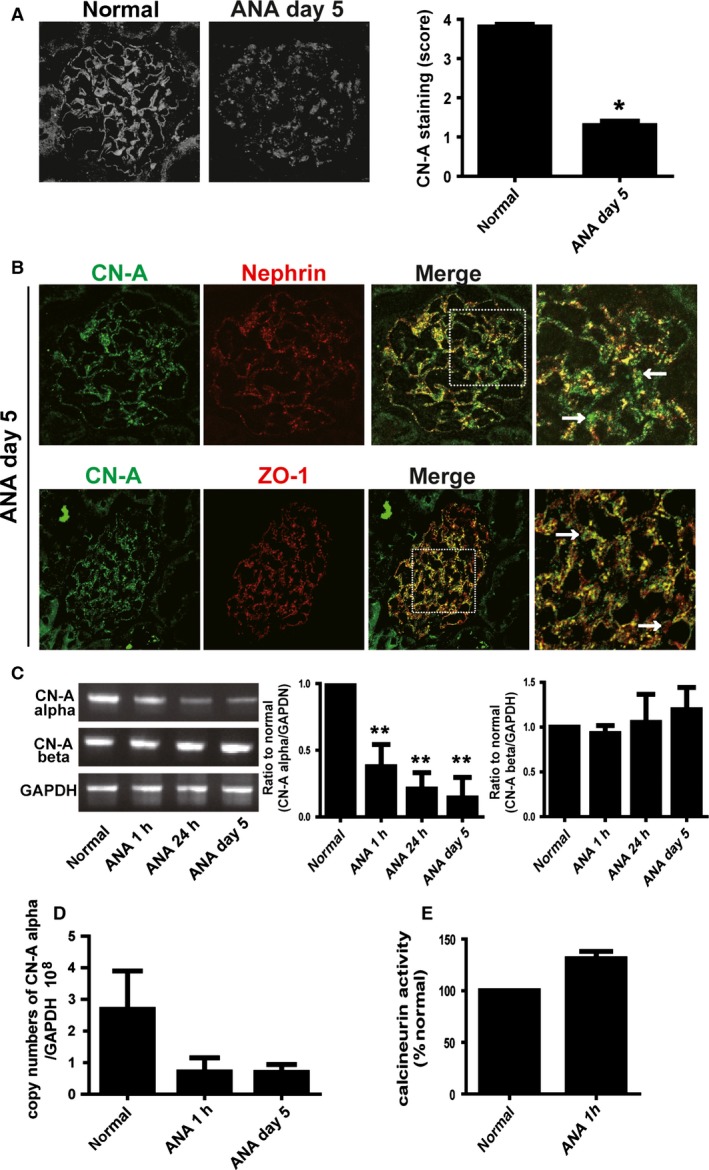
The expression and the activity of CN‐A are altered in anti‐nephrin antibody (ANA)‐induced nephropathy. (A) In ANA‐induced nephropathy, the staining pattern of CN‐A changed to a discontinuous fine granular pattern, and the staining intensity was decreased on day 5, when proteinuria peaked. Data from the semiquantitative evaluation of the CN‐A staining are shown in the right panel. The score is shown as mean ± SD (*P <* *0.05 vs. normal control). (B) Upper panel: Dual‐labeling IF findings of CN‐A (green) with nephrin (red) on day 5 of ANA‐induced nephropathy. Major portions of the remaining CN‐A staining were dissociated from the nephrin staining (detected as green, arrows), although the remaining nephrin was still colocalized with CN‐A (detected as yellow). (B) Lower panel: Dual‐labeling IF findings of CN‐A (green) with ZO‐1 (red). The stainings of CN‐A and ZO‐1 were dissociated (arrows). (C) The representative electrophoretic pattern of semiquantitative RT‐PCR findings of CN‐A‐*α* and ‐*β* is shown in the left panel. mRNA expression of CN‐A‐*α* was clearly decreased at 1 and 24 h and on day 5 after ANA injection (**P <* *0.0005 vs. normal). In contrast, mRNA expression of CN‐A‐*β* was not altered in any time points in ANA nephropathy. (D) The decrease in the mRNA expression of CN‐A‐*α* was confirmed by the real‐time RT‐PCR analysis with a standard of the plasmid containing a cDNA of CN‐A‐*α*. The copy numbers of mRNA for CN‐A‐*α* evaluated with real‐time RT‐PCR are shown. (E) The phosphatase activity of CN in glomerular lysates was evaluated by the CN activity assay kit. The CN activity was promoted at 1 h of ANA‐induced nephropathy.

The immunostaining of CN‐A shifted to a discontinuous pattern from the early phase (1 and 24 h), and the staining intensity of CN‐A was clearly decreased on day 5 of anti‐nephrin antibody‐induced nephropathy, when proteinuria peaked (Fig. 5A). The staining score was clearly decreased on day 5 (*P* < 0.05 vs. normal control). Dual‐labeling analyses with nephrin and ZO‐1 showed that the major portions of the remaining CN‐A staining was dissociated from nephrin and ZO‐1 (Fig. 5B). mRNA expression of CN‐A in the anti‐nephrin antibody‐induced nephropathy was analyzed by semiquantitative RT‐PCR of the glomerular cDNA with specific primers for CN‐A‐*α* and CN‐A‐*β*. The results are shown in Figure 5C. The mRNA expression of CN‐A‐*α* was clearly decreased at 1 and 24 h, and the decrease was still detected on day 5 (*P* < 0.0005 vs. normal). In contrast, mRNA expression of CN‐A‐*β* was not altered in any time points. The decrease in mRNA expression of CN‐A‐*α* was confirmed by real‐time RT‐PCR analysis with a standard of the plasmid containing a cDNA of CN‐A‐*α*. The copy number of mRNA for CN‐A‐*α* was clearly decreased (Fig. 5D). The phosphatase activity of CN in glomeruli was promoted at 1 h of the anti‐nephrin antibody‐induced nephropathy (Fig. 5E), and the increase was still detected on day 5.

### Tacrolimus ameliorated proteinuria and the redistribution of CN‐A and nephrin in anti‐nephrin antibody‐induced nephropathy

**Figure 6:**
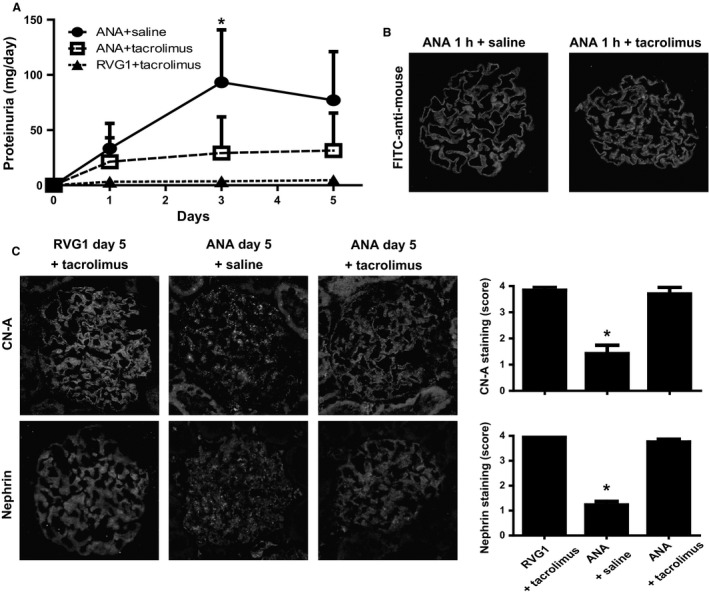
Tacrolimus ameliorated proteinuria and the redistribution of CN‐A and nephrin in anti‐nephrin antibody (ANA)‐induced nephropathy. (A) Tacrolimus treatment significantly reduced the amount of proteinuria on day 3 (29.2 vs. 93.3 mg/day, *P <* *0.05). RVG1: irrelevant murine IgG1. (B) The distribution of the ANA injected in glomeruli was detected with FITC‐anti‐mouse IgG as a continuous linear pattern along the capillary loop. No differences in the staining of the injected ANA were observed between rats with and without tacrolimus treatment. (C) The tacrolimus treatment inhibited the alteration of the stainings of CN‐A and nephrin on day 5. The representative IF findings are shown in the left panel. Semiquantitative evaluation showed that the stainings of the CN‐A and nephrin were preserved in rats with tacrolimus treatment (right panel). The score is shown as mean ± SD (*P <* *0.05 vs. ANA + saline).

The effect of tacrolimus on proteinuria in anti‐nephrin antibody‐induced nephropathy was investigated. The kinetics of proteinuria of rats treated with tacrolimus (0.3 mg/kg BW) is shown in Figure 6A. The tacrolimus treatment significantly ameliorated proteinuria on day 3 (29.2 vs. 93.3 mg/day, *P* < 0.05). No differences in the staining of the anti‐nephrin antibody injected, which was detected with FITC‐anti‐mouse IgG, were observed between rats with and without tacrolimus treatment, indicating that the tacrolimus treatment did not affect the distribution of the anti‐nephrin antibody (Fig. 6B). The tacrolimus treatment inhibited the alterations of the stainings of CN‐A and nephrin on day 5. The representative IF finding and the data of the semiquantitative analysis are shown in Figure 6C.

### CN‐A staining in podocytes is altered in a human case with nephrotic syndrome

**Figure 7:**
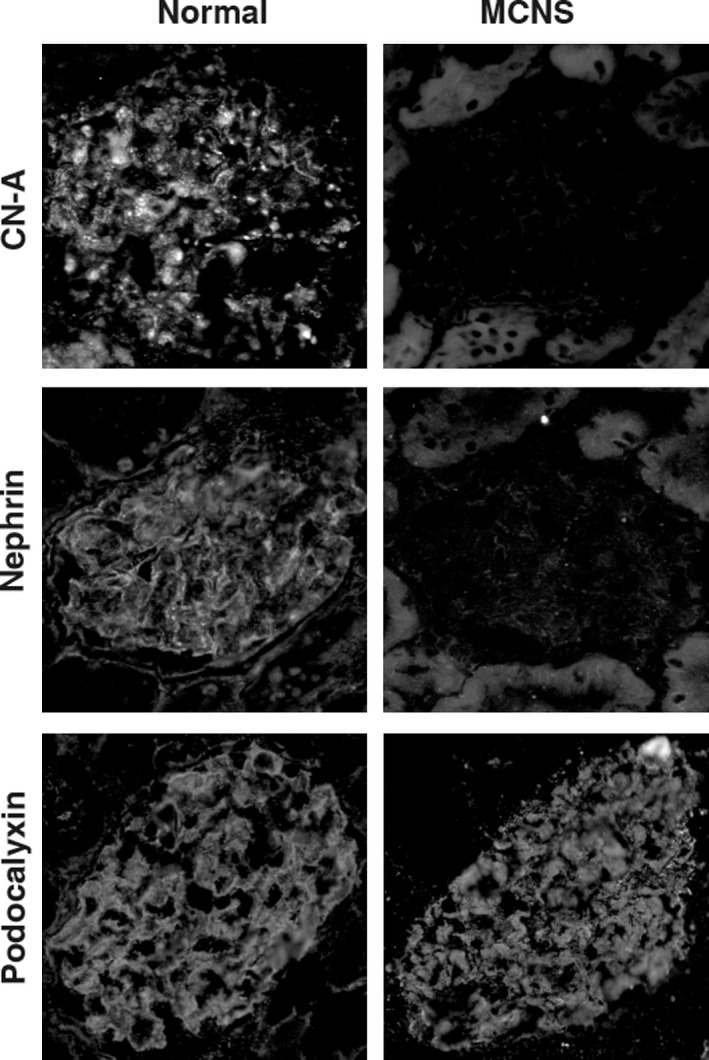
Expression of the CN‐A in podocyte is altered in a human case with nephrotic syndrome. The positive stainings of the CN‐A, nephrin, and podocalyxin in podocytes were detected in the control human kidney section. The stainings of CN‐A and nephrin were clearly reduced in the kidneys of a 10‐year‐old boy who had relapsing steroid‐resistant nephrotic syndrome, whereas podocalyxin staining was not altered.

Positive stainings of CN‐A, nephrin, and podocalyxin were detected in the podocytes of control human kidney sections (Fig. 7). The stainings of CN‐A and nephrin were clearly reduced in the kidneys of a 10‐year‐old boy who had relapsing steroid‐resistant nephrotic syndrome, whereas podocalyxin staining was not altered.

## Discussion

CN, a Ca^2+^ and calmodulin‐dependent protein serine/threonine phosphatase, is widely distributed in many cell types and understood to play an important role in the functioning of the cells. Although CN is reported to be highly distributed in the kidneys (Rusnak and Mertz 2000; Shibasaki et al. 2002), the role of CN in the kidneys is not yet fully understood. In the present study, we investigated the expression and the role of CN‐A in glomeruli. First, the localization of CN‐A in glomeruli was analyzed with the specific antibody that recognizes a full‐length endogenous form of CN‐A‐*α* and ‐*β* (Talmadge et al. 2004). An approximately 60 kDa band corresponding to the full‐length CN‐A was detected in normal rat glomerular lysates by Western blot analysis. In the immunohistological analyses, clear staining was observed along the glomerular capillary wall in normal rat and human kidney sections (Fig. 1). Because the staining in glomeruli was abolished by preabsorption with a fusion protein of CN‐A (Fig. 1A), it was concluded that the staining is a specific one.

Dual‐labeling immunohistological studies with glomerular cell markers showed that CN‐A is exclusively expressed in podocytes. To further analyze the subcellular localization of CN‐A in podocyte, the dual‐labeling studies with podocyte markers were done. The studies showed that CN‐A is dominantly expressed at the basal side of the podocyte and that CN‐A is colocalized with nephrin and ZO‐1, critical components of the SD (Fig. 2). Duolink analysis (Soderberg et al. 2006; Fukusumi et al. 2015) showed the close localization of CN‐A and nephrin within 40 nm (Fig. 3A). Nephrin is detected as double bands in the whole glomerular lysate (Simons et al. 2001), and the upper band corresponds to an N‐glycosylated membrane form of nephrin which is localized at the SD (Yan et al. 2002). In this study, an upper band was densely detected in the precipitate with the anti‐CN‐A antibody (Fig. 3B). Besides, not only nephrin but also other SD components, ZO‐1 and podocin, were detected in the anti‐CN‐A precipitate. These observations showed that CN‐A is a member of the molecular complex constituting the SD. The specific binding partner of CN‐A in the SD complex has not been determined in this study. However, because ZO‐1 has a CN docking motif LXVP (LNVP: aa 257–260), it is supposed that CN‐A binds to ZO‐1 at this motif. It is conceivable that CN‐A interacts with nephrin via ZO‐1 and NEPH1, because it is reported that ZO‐1 binds to NEPH1 and NEPH1 binds to nephrin (Huber et al. 2003; Liu et al. 2003).

In this study, the expression of CN in developing glomeruli was also analyzed. The CN‐A staining at the presumptive podocyte was first detected at the late S‐shaped body stage, when nephrin first appeared (Kawachi et al. 1995). The clear CN‐A staining was detected in the early capillary loop stage and was colocalized with nephrin (Fig. 4). These observations also showed that CN‐A is highly associated with nephrin. CN‐A might be involved in the maturation of the SD.

To elucidate the functional association of CN with nephrin and other SD molecules, the expression of CN‐A was analyzed in the nephrotic model caused by the antibody against nephrin. We have previously reported that the expressions of not only nephrin but also other SD molecule such as ZO‐1 and NEPH1 were altered from the early phase of this nephrotic model (Kawachi et al. 1997, 2000; Miyauchi et al. 2006). The present study revealed that the immunostaining of CN‐A shifted to a discontinuous pattern from the early phase and its staining intensity was clearly decreased on day 5 of this nephrotic model similar to these SD molecules (Fig. 5A). It is known that CN‐A has two isoforms of CN‐A‐*α* and CN‐A‐*β*. In this study, we found that although mRNA expression of CN‐A‐*α* was decreased, any changes in the mRNA expression οf CN‐A‐*β* were not detected (Fig. 5C). These observations suggested that not CN‐A‐*β* but CN‐A‐*α* is a component of the SD components and that the alteration in the expression of CN‐A‐*α* participates in development of proteinuria in this model.

Unexpectedly, in this study, we found that the phosphatase activity of CN of glomerular lysate was increased in the anti‐nephrin antibody‐induced nephropathy (Fig. 5E), although the expressions of CN‐A‐*α* were decreased in this nephrotic model. Although it is difficult to explain the etiological relationship of these findings well, it is plausible that the activated CN‐A turns to be hard to be detected by the antibody because of the changes of its subcellular localization and/or of its molecular conformation. It may be possible to explain the findings with the following hypothesis that the CN‐A retained at the SD is an inactive form, and the activated CN‐A released from the SD to cytoplasm changes to be hard to be detected by the antibody. mRNA expression of CN‐A‐*α* decreases as a negative feedback response against the increase of the phosphatase activity of CN.

In this study, next we elucidated the pathogenic significance of the increase of the CN activity in this nephrotic model. We investigated the effect of the CNI tacrolimus on proteinuria and redistribution of the SD molecules in this nephrotic model. We found that the tacrolimus treatment ameliorated proteinuria (Fig. 6A). It is also observed that the tacrolimus treatment suppressed the redistribution of CN‐A and nephrin (Fig. 6C). These observations indicate that the increase of CN activity participates in the development of the SD injury of this nephrotic model. Although the mechanism how the activation of CN‐A causes podocyte injury is unclear, the podocyte injury seems to be caused not via synaptopodin, because any alterations in the expression of synaptopodin was detected in this nephrotic model. Although the precise pharmacological mechanism are unclear, the results in this study showed that CNI has a direct effect on podocyte, because the dysfunction of the SD of this model was confirmed to be caused independently with any inflammatory factors (Kawachi et al. 2002) or hemodynamic changes (Kawachi et al. 2002; Suzuki et al. 2007).

In this study, we also analyzed the expression of CN‐A in clinical biopsy samples. We observed that the expression of the CN‐A at the SD is clearly decreased in a patient with active MCNS (Fig. 7). Although further studies with more cases are necessary, the finding of this case may suggest that the decrease in the expression of CN‐A at the SD participates in the development of proteinuria in MCNS. Monitoring the expression of CN‐A in the biopsy materials may help to recognize the podocyte injury related with CN dysregulation and the sensitivity to the CNI treatment.

In conclusion, the present study showed that an endogenous form of CN‐A is distributed at the SD and that CN‐A is highly associated with nephrin, a critical component of the SD. The phosphatase activity of CN in glomeruli was elevated in rat nephrotic model caused by the antibody against nephrin, although the expression of CN‐A at the SD is decreased. We showed here that the CNI tacrolimus ameliorated proteinuria in the nephrotic model by inhibiting the redistribution of CN‐A and nephrin. The results suggest that CNI can be an effective medicine for patients with nephrotic syndrome caused by the SD dysfunction.

## Conflict of Interest

None declared.
